# Alteration of gut microbiome and correlated amino acid metabolism are associated with acute myelocytic leukemia carcinogenesis

**DOI:** 10.1002/cam4.6283

**Published:** 2023-07-06

**Authors:** Jing Xu, Yong Kang, Yan Zhong, Wencan Ye, Tianle Sheng, Qingming Wang, Jifu Zheng, Qiuyue Yang, Ping Yi, Zhenjiang Li

**Affiliations:** ^1^ Department of Hematology The Second Affiliated Hospital of Nanchang University Nanchang China; ^2^ Department of Hematology First Affiliated Hospital of Gannan Medical University Ganzhou China; ^3^ Department of General Medicine Ganzhou People's hospital Ganzhou China; ^4^ Department of Clinical Laboratory The Second Affiliated Hospital of Nanchang University Nanchang China; ^5^ Department of Scientific Research Project Wuhan Kindstar Medical Laboratory Co., Ltd. Wuhan China; ^6^ Kindstar Global Precision Medicine Institute Wuhan China

**Keywords:** acute myelocytic leukemia, amino acids, fecal metabolomics, gut microbiota

## Abstract

**Background:**

The aim of this study is to investigate the profiles of gut microbiota and metabolites in acute myelocytic leukemia (AML) patients treated with/without chemotherapy.

**Methods:**

Herein, high‐throughput 16S rRNA gene sequencing was performed to analysis gut microbiota profiles, and liquid chromatography and mass spectrometry were performed to analysis metabolites profiles. The correlation between gut microbiota biomarkers identified by LEfSe and differentially expressed metabolites were determined by spearman association analysis.

**Results:**

The results showed the distinguished gut microbiota and metabolites profiles between AML patients and control individuals or AML patients treated with chemotherapy. Compared to normal populations, the ratio of *Firmicutes* to *Bacteroidetes* was increased at the phylum level than that in AML patients, and LEfSe analysis identified *Collinsella* and *Coriobacteriaceae* as biomarkers of AML patients. Differential metabolite analysis indicated that, compared to AML patients, numerous differential amino acids and analogs could be observed in control individuals and AML patients treated with chemotherapy. Interestingly, spearman association analysis demonstrated that plenty of bacteria biomarkers shows statistical correlations with differentially expressed amino acid metabolites. In addition, we found that both *Collinsella* and *Coriobacteriaceae* demonstrate remarkable positive correlation with hydroxyprolyl‐hydroxyproline, prolyl‐tyrosine, and tyrosyl‐proline.

**Conclusion:**

In conclusion, our present study investigated the role of the gut‐microbiome–metabolome axis in AML and revealed the possibility of AML treatment by gut‐microbiome–metabolome axis in the further.

## 1 INTRODUCTION

Acute myelocytic leukemia (AML) is a clinical and genetic heterogeneous hematologic malignancy with high mortality, characterized by the blocked normal differentiation of progranulocytes or the proliferation of immature myeloid progenitors.^1^, ^2^, ^3^ Chemotherapy with cytarabine and anthracycline in combination with or without allogeneic hematopoietic cell transplantation considered as its standard therapeutic strategy.^4^, ^5^ With the more uniform practice of stem cell transplantation and emergence of novel agents, the survival time of AML patients have improved remarkably. However, approximately 30% of patients are insensitive to routine chemotherapy, and at least 50% of those achieved remission will relapse, resulting in worse prognosis.^5^, ^6^ Thus, it is of great imperious to investigate the pathogenicity of AML patients to develop effective therapeutic strategy.

Numerous studies have demonstrated that gut microbiota is involved in the pathogenesis of multiple diseases, such as nonalcoholic fatty liver,^7^ gastrointestinal cancer,^8^ and hematological malignancies.^9^ Therefore, attention toward microbiota‐based cancer therapies has been mounting. Clinical studies have demonstrated the effectiveness of fecal microbiota transplantation or dietary interventions to improve the success rate of therapy in patients with cancer.^10^ Chemotherapy changes the diversity and abundance of gut microbiota in patients, and gut microbiota is considered as a potential candidate to predict the outcome of chemotherapy and prevent adverse events.^11^ Moreover, accumulating research have revealed that the metabolites such as peptidoglycans and short‐chain fatty acids generated by the gut microbiota keep local immunity in the intestinal region.^12^ In addition, evidence has revealed that there exist strong correlations between stool metabolites and microbiome in patients with allogeneic hematopoietic cell transplantation, which provides reference for follow‐up intervention and treatment.^13^

In recent years, increasing populations devote to explore the effect of the gut microbiota in pathological change of hematological malignancies. Prvious study have revealed the remarkable reduce of gut microbiota diversity in AML patients with febrile neutropenia after intensive chemotherapy.^14^ However, the relationship between intestinal microbiome and metabolites as well as their correlation in AML patients are barely elucidated. Herein, we analyzed the diversity and abundance of intestinal microflora and differential metabolites of AML patients treated with/without chemotherapy using 16s rRNA and liquid chromatography and mass spectrometry (LC–MS), respectively, and the correlation between them was analyzed.

## 2 MATERIALS AND METHODS

### 2.1 Patients and treatment

A total of 11 newly diagnosed AML patients and 21 age and sex matched healthy individuals or healthy adults that live with patients (termed as CON group) from January 2020 to December 2020 in The Second Affiliated Hospital of Nanchang University were enrolled in the study. Patients with gastrointestinal resection, inflammatory bowel disease, irritable bowel syndrome, chronic constipation, probiotics administration within 3 months before sampling, secondary AML, antecedent myelodysplastic syndrome, or acute promyelocytic leukemia were excluded. Morphological, molecular analyses, cytogenetic, and, flow cytometric were performed at diagnosis, and patients were diagnosed as AML according to the World Health Organization (2016) classification.^15^ The baseline characteristics of all enrolled patients are shown in Table 1. Total of 11 newly diagnosed AML patients before treatment were termed as AML_N1 group, 5 patients among AML_N1 group after 7 days of traditional chemotherapy treatment (DA or IA regimen) were termed as AML_N2 group. The other six patients who had received antibiotics prior to chemotherapy were excluded. The stools of patients before and after 7 days of treatment were collected and maintained under −80°C for the subsequent microbiota analysis. Furthermore, the stool samples of AML_N2 group and five randomly selected samples in both AML_N1 and CON groups were subjected to metabolome analysis. This study was approved by the Ethics Committee of The Second Affiliated Hospital of Nanchang University (Approved number: 2018–098) and all the patients were informed consent.

**TABLE 1:** Clinical and genetic characteristics of AML patients.

Characteristic	AML patients
Age (median, range)	57.2 (18–73)
Sex (*n*, %)	
Male	6 (55%)
Female	5 (45%)
WBCs (×10^9^/L, median, range)	12.6 (1.1–34.2)
HB (g/dL, median, range)	84.6 (61–128)
PLTs (×10^9^/L, median, range)	44.5 (5–151)
BM blast (median, range)	58.5% (38–92%)
Cytogenetic risk (*n*, %)	
Favorable	3 (27%)
Intermediate	5 (45%)
Unfavorable	2 (18%)
Missing	1 (9%)
Mutation (*n*, %)	
NRAS	1 (9%)
TP53	1 (9%)
CEBPA	3 (27%)
TET2	3 (27%)
IKZF1	1 (9%)
IDH1	1 (9%)
None	5 (45%)
Missing	1 (9%)

Abbreviations: BM: bone marrow; HB, hemoglobin level; PLT, Platelet; WBCs, white blood cells count.

### 2.2 High‐throughput 16S rRNA gene sequencing and data analysis

The high‐throughput 16S rRNA gene sequencing was supported by Shanghai Oebiotech Biotechnology Co., Ltd. Briefly, total DNA from intestinal digesta was extracted using MagPure Soil DNA LQ Kit (Magen) and quantified using NanoDrop 2000 (Thermo Fisher Scientific). The V3‐V4 regions of the bacterial 16S rRNA gene were amplified using Tks Gflex DNA Polymerase (Takara) with universal primers (with the barcode) as follow sequences: Forward, 5’‐TACGGRAGGCAGCAG‐3′, reverse, 5’‐AGGGTATCTAATCCT‐3′.^16^ The sequencing libraries were generated and sequenced on an Illumina NovaSeq PE250 platform. Raw sequences quality control and demultiplex were performed using the Quantitative Insights Into Microbial Ecology (QIIME) software (version 1.8.0).^17^ Trimmomatic software was used to remove clutter from the original paired‐end sequences.^18^ The obtained sequences were then assembled using FLASH software.^19^ Only reads with overlap longer than 10 bp were assembled, whereas the unassembled sequences were discarded. Operational taxonomic units (OTUs) were clustered using Vsearch software with 97% similarity cutoff,^20^ and Chimeric sequences were identified and removed using UCHIME. The representative read of each OTU was selected using QIIME package. All representative reads were annotated and blasted against Silva database (Version 138) using RDP classifier (confidence threshold was 70%).^21^ Alpha (α) diversity indexes, such as Chao1, Shannon, goods coverage, observed species, PD whole, and Simpson, were performed to evaluate diversity and richness of OTUs among samples. Beta (β)‐diversity analysis was performed to identify the microbial communities' structural variations among groups by principal coordinate analysis (PCoA) and nonmetric multidimensional scaling (NMDS) based on the Bray–Curtis distance. In addition, linear discriminate analysis (LDA) effect size (LEfSe) analysis was performed to identify the species that had significant differences in sample classification. Data were computed with an LDA score above 2.00 and *p <* 0.05 for the factorial Kruskal–Wallis test.

### 2.3 Liquid chromatography–mass spectrometry‐based metabolome analysis and data processing

LC–MS‐based metabolome analysis was commercial performed by Shanghai Lu‐Ming Biotech Co. Ltd. In detail, 60 mg of intestinal contents of each sample was added to 20 μL internal standard L‐2‐chlorophenylalanine (0.3 mg/mL, Shanghai Heng chuang Bio‐technology Co., Ltd.) and 600 μL of 80% methyl alcohol. After treatment, 150 μL of the supernatant was collected and transferred into LC vials, and subjected to LC–MS analysis. Quality control samples were prepared by mixing aliquot of the whole samples to be a pooled sample. LC–MS analysis was performed using a Dionex Ultimate 3000 RS UHPLC fitted with Q‐Exactive plus quadrupole‐Orbitrap mass spectrometer equipped with heated electrospray ionization (ESI) source (Thermo Fisher Scientific). The column was ACQUITY UPLC HSS T3 (1.8 μm, 2.1 × 100 mm). System parameters were as follows: mobile phase A: water containing 0.1% formic acid (v/v); mobile phase B: acetonitrile containing 0.1% formic acid (v/v); elution gradient: 0 min, 5% B; 2 min, 5% B; 4 min, 25% B; 8 min, 50% B; 10 min, 80% B; 14 min, 100% B; 15 min, 100% B; 15.1 min, 5% and 16 min, 5%B; column temperature: 45°C; flow rate: 0.35 mL/min; injection volume: 2 μL; sample temperature: 4°C; mass range: 100–1000 m/z; spray voltage: 3800 V (ESI+) and 3000 V (ESI‐); capillary temperature: 320°C; aux gas heater temperature: 350°C; sheath gas flow rate, 35 arbitrary units (arb); auxiliary gas flow rate: 8 arb; S‐lens RF level: 50.

The original LC–MS data were normalized using Progenesis QI V2.3 software. Compound identification was performed using The Human Metabolome Database, Lipidmaps (V2.3), Metlin, Electron Microscopy Data Bank, PMDB, and self‐built databases. Multivariate statistical analysis was performed by unsupervised principal component analysis to observe the overall distribution between samples and the stability of the whole analysis process, followed by supervised orthogonal partial least‐squares‐discriminant analysis (OPLS‐DA) to distinguish the differential metabolites among groups. Fold change (FC) analysis and Student's *t*‐test were utilized to detect the fold change and statistical significance of per metabolite among groups. Variable importance of projection (VIP) values obtained from the OPLS‐DA model were used to rank the overall contribution of each variable to group discrimination. Metabolites with FC ≥2, VIP ≥1, *p* < 0.05 were considered as differential metabolites. Volcano plots were visualized using R package based on log10 (*p*‐value) and log2 (FC).

### 2.4 Statistical Analysis

Referred to previous studies^14^, ^22^, ^23^ and the fact that it is difficult to collect newly diagnosed AML patients without antibiotics treatment due to the newly diagnosed AML patients are often complicated with infection and fever, only a total of 11 newly diagnosed AML patients and 21 matched healthy controls were enrolled in this study. Enumeration data were expressed as frequency and percentage (%), and measurement data are shown as mean ± SD. Statistic difference in biological parameters among groups were determined using Wilcoxon rank sum test or Kruskal–Wallis test. The correlation between significant difference gut bacteria in sample classification and differential amino acids and analogs was evaluated using Spearman association analysis. All analyses were conducted using R software (2.5–6 vegan). A *p* < 0.05 was considered as statistical significance.

## 3 RESULTS

### 3.1 The change of gut microbiota in AML patients treated with/without chemotherapy

**FIGURE 1:**
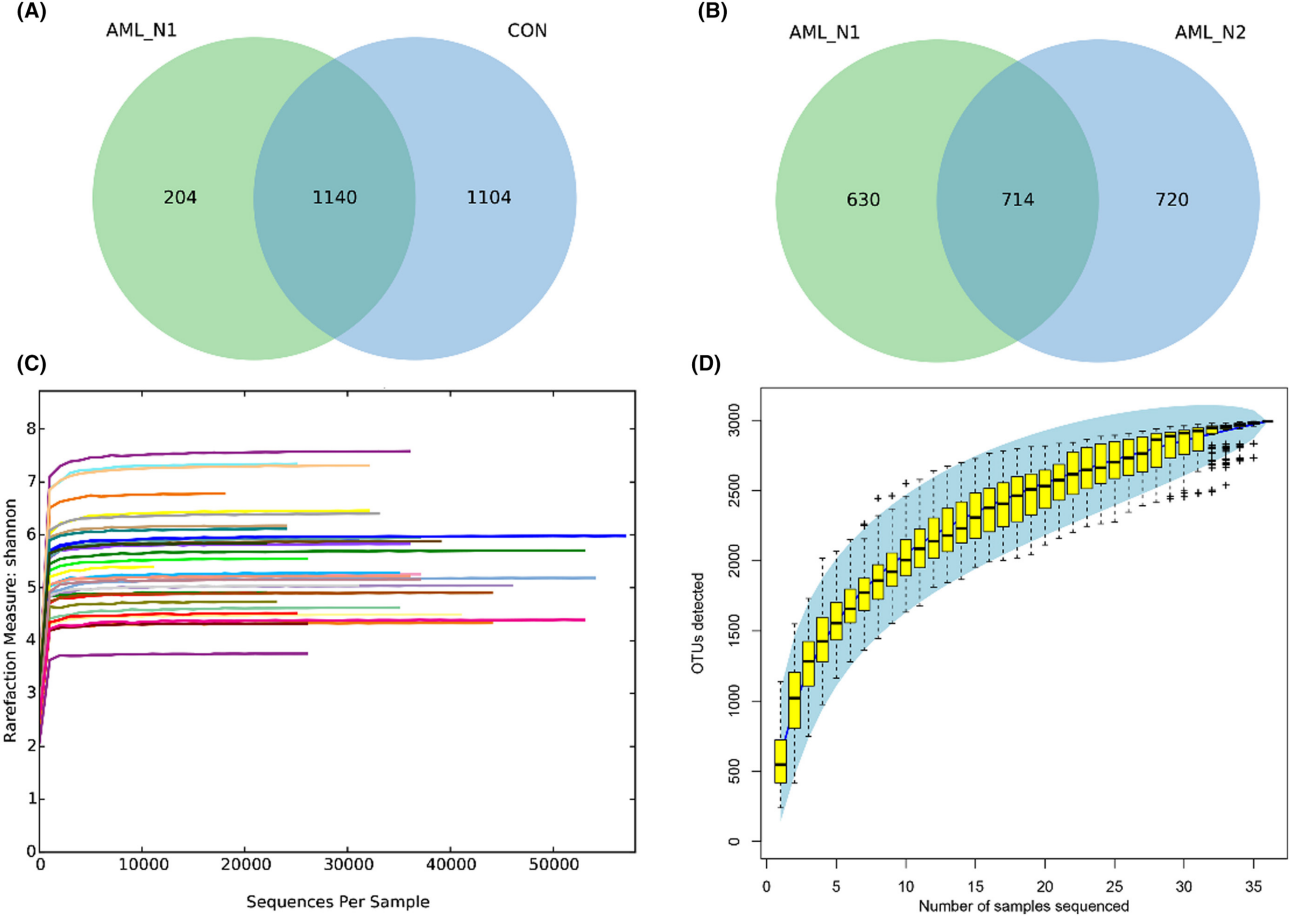
Adequate sequencing and sampling. (A) Venn diagram shows the unique and shared OTUs in AML_N1 and CON groups. (B) Venn diagram shows the unique and shared OTUs in AML_N1 and AML_N2 groups. (C) The rarefaction curves of Shannon index of each sample. (D) Specaccum curve of all fecal samples. CON group, Normal control; AML_N1 group, newly diagnosed AML patients; AML_N2 group, Newly diagnosed AML patients treated with chemotherapy.

**FIGURE 2:**
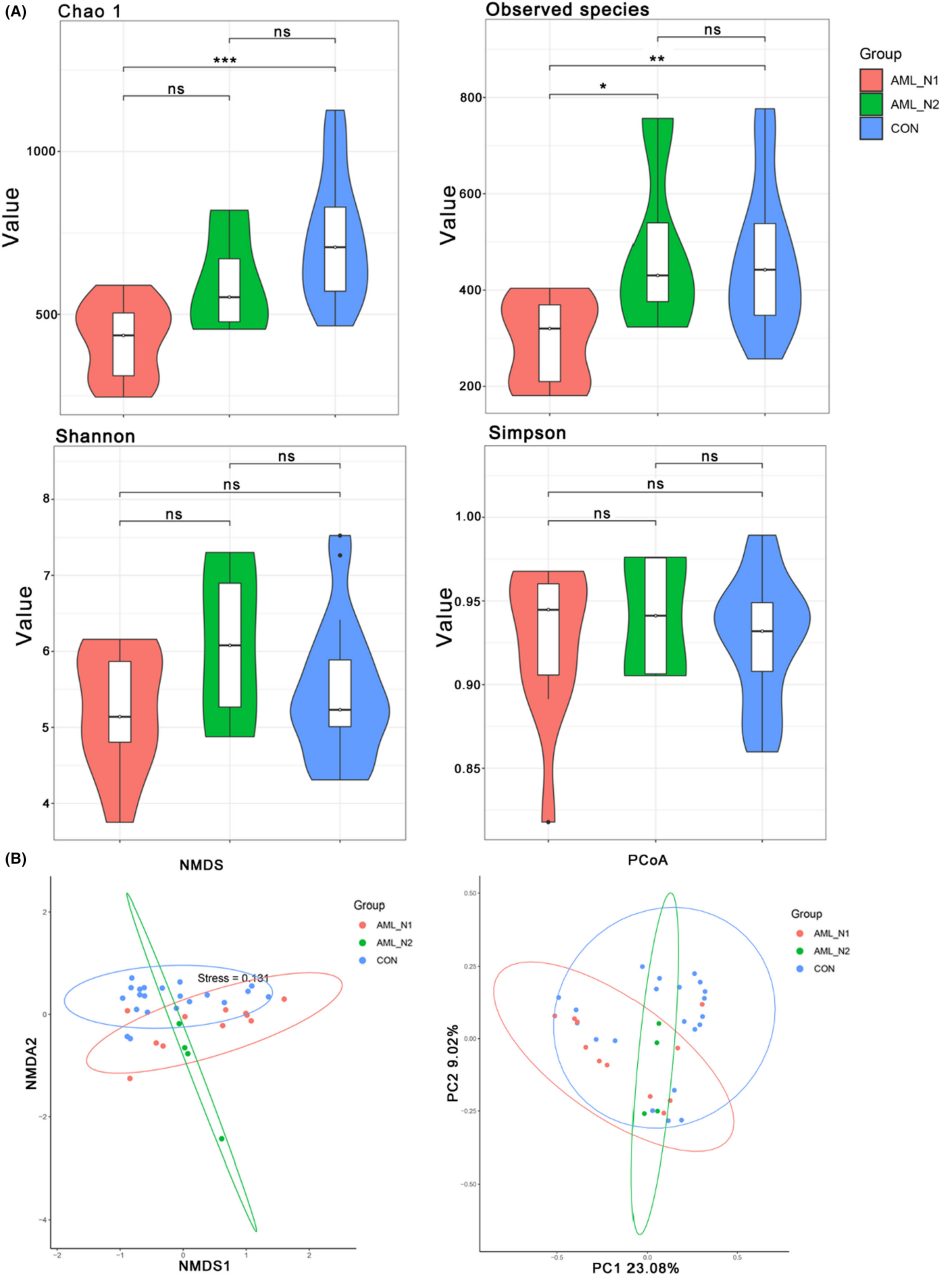
Microbial diversity of fecal samples. (A) α‐diversity showed by the box plot of the Chao1 index, Observed species index, Shannon index, and Simpson index among groups. (B) β‐Diversity presented by the NMDS and PCoA plot of per sample based on Bray‐Curtis metrics. CON group, normal control; AML_N1 group, newly diagnosed AML patients; AML_N2 group, newly diagnosed AML patients treated with chemotherapy. *p < 0.05, **p < 0.01, ***p < 0.001.

Gut microbiota of each group were explored by detecting the V3‐V4 regions of the 16S rRNA gene. A total of 1,146,660 clean reads were obtained from AML_N1 and CON samples, with a mean of 35,833 ± 9716 reads in each sample. These clean reads were clustered into 2448 OTUs by the standard of 97% similarity cutoff, among which 1140 OTUs were shared in twos (overlap area of the Venn diagram), 204 unique OTUs were observed in AML_N1 group, and 1104 unique OTUs exhibited in CON group (Figure 1A). Whereas a total of 396,897 clean reads were obtained from AML_N1 and AML_N2 samples, with a mean of 26,457 ± 7926 reads in each sample. These clean reads were clustered into 2064 OTUs by the standard of 97% similarity cutoff, among which 714 OTUs were shared in twos, 630 unique OTUs were detected in AML_N1 group, and 720 unique OTUs exhibited in AML_N2 group (Figure 1B). The rarefaction curves (Figure 1C) and Specaccum curve (Figure 1D) were gradually level off with the increasing of sequences and samples number, respectively, indicating the adequate of sequencing and sampling. α‐Diversity analysis was conducted to determine the abundance and diversity of species.^24^ As shown in Figure 2A, compared to normal population (CON group), the Chao1 index and observed species index (proxies for community richness) were decreased in newly diagnosed patients (AML_N1group), and the observed species index of patients in AML_N2 group was increased compared to that in AML_N1 group. Whereas, the Shannon index and Simpson index (proxies for community diversity, considering both richness and evenness) exhibited non‐significant trend toward a slight reduction in newly diagnosed AML patients compared to that in patients treated with chemotherapy and control individuals. Furthermore, β‐diversity presented by the NMDS and PCoA plot of per sample based on Bray–Curtis metrics revealed the distinguished microbiota profiles among groups (Figure 2B).

**FIGURE 3:**
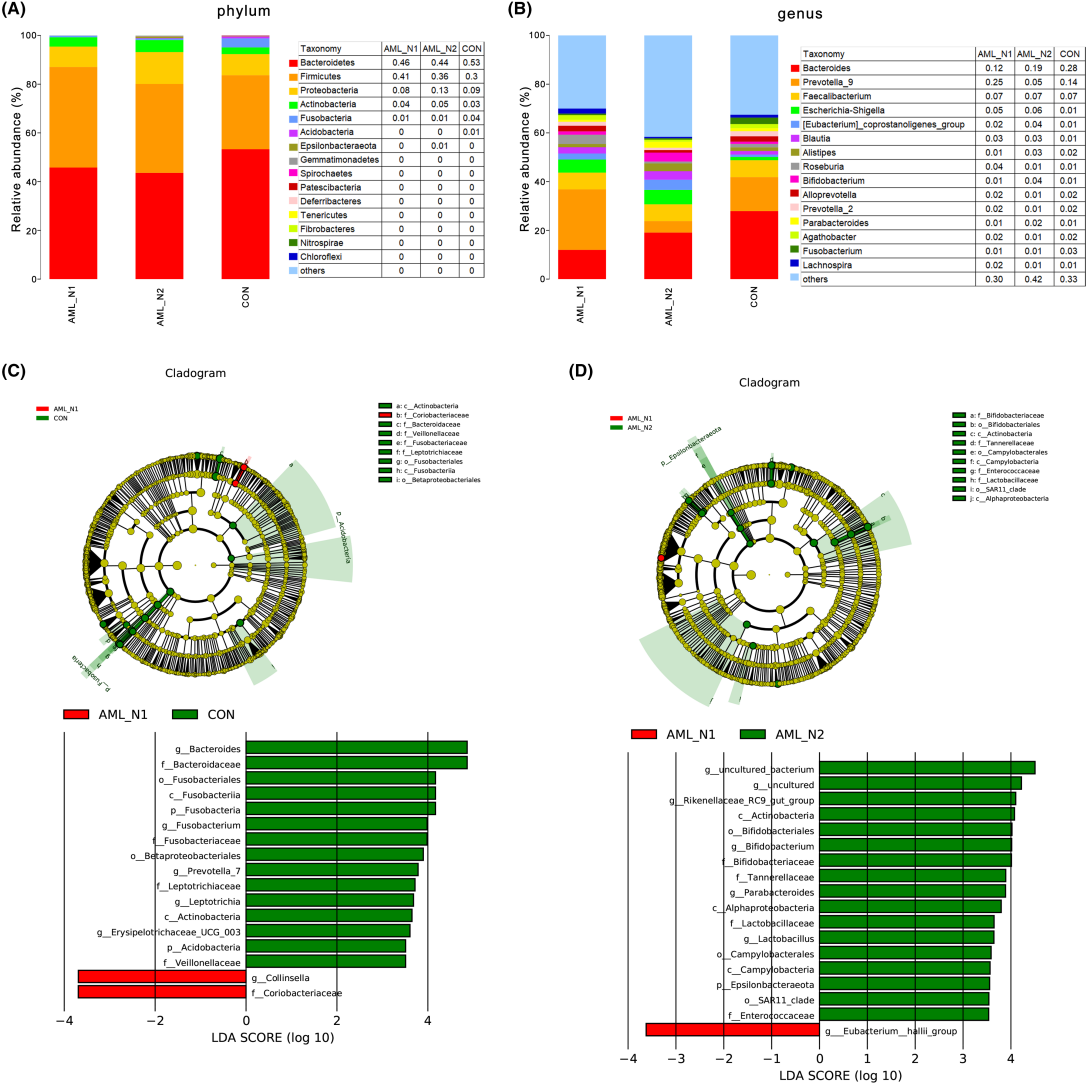
Intestinal microbiota pattern and differential microbe in each group. (A) Constituent and abundance of top 15 gut microbiotas at phylum in each group. (B) Constituent and abundance of top 15 gut microbiotas at genus levels in each group. (C) The cladogram and differential microbe analyzed by LEfSe in AML_N1 and CON groups. (D) The cladogram and differential microbe analyzed by LEfSe in AML_N1 and CON groups. CON group, normal control; AML_N1 group, Newly diagnosed AML patients; AML_N2 group, Newly diagnosed AML patients treated with chemotherapy.

Microbial structure abundance and relative quantification at phylum and genus levels are shown in Figure 3A,B, respectively, revealing the obvious distinction of the community richness among these three groups. *Bacteroidetes*, *Firmicutes*, and *Proteobacteria* were the top three dominated community at phylum level, contributing to 92% in CON group, 95% in AML_N1 group, and 93% in AML_N2 group. The ratio of *Firmicutes* to *Bacteroidetes* was remarkable increased in newly diagnosed AML patients compared with that in control individuals, which was slightly rescued by chemotherapy. *Bacteroides*, *Prevotella_9* and *Faecalibacterium* were the major community at genus level. To identify specific bacteria in each group, LEfSe analysis was performed and the corresponding cladogram were generated, suggesting that there were several bacteria characterized as biomarkers between groups. As shown in Figure 3C, *Collinsella* and *Coriobacteriaceae* were significantly enriched in newly diagnosed AML patients compared with that in control individuals, whereas *Bacteroides*, *Bacteroidaceae*, *Fusobacteriales*, *Fusbacteria*, etc. were enriched in healthy controls (Figure 3C). Compared with patients in AML_N1 group, several bacteria, such as *Actinobacteria*, *Bifidobacterium*, *Bifidobacteriales*, etc., were enriched in patients of AML_N2 group, whereas Eubacterium_hallii_group was enriched in patients of AML_N1 group (Figure 3D).

### 3.2 The change of metabolite profiles in AML patients treated with/without chemotherapy

**FIGURE 4:**
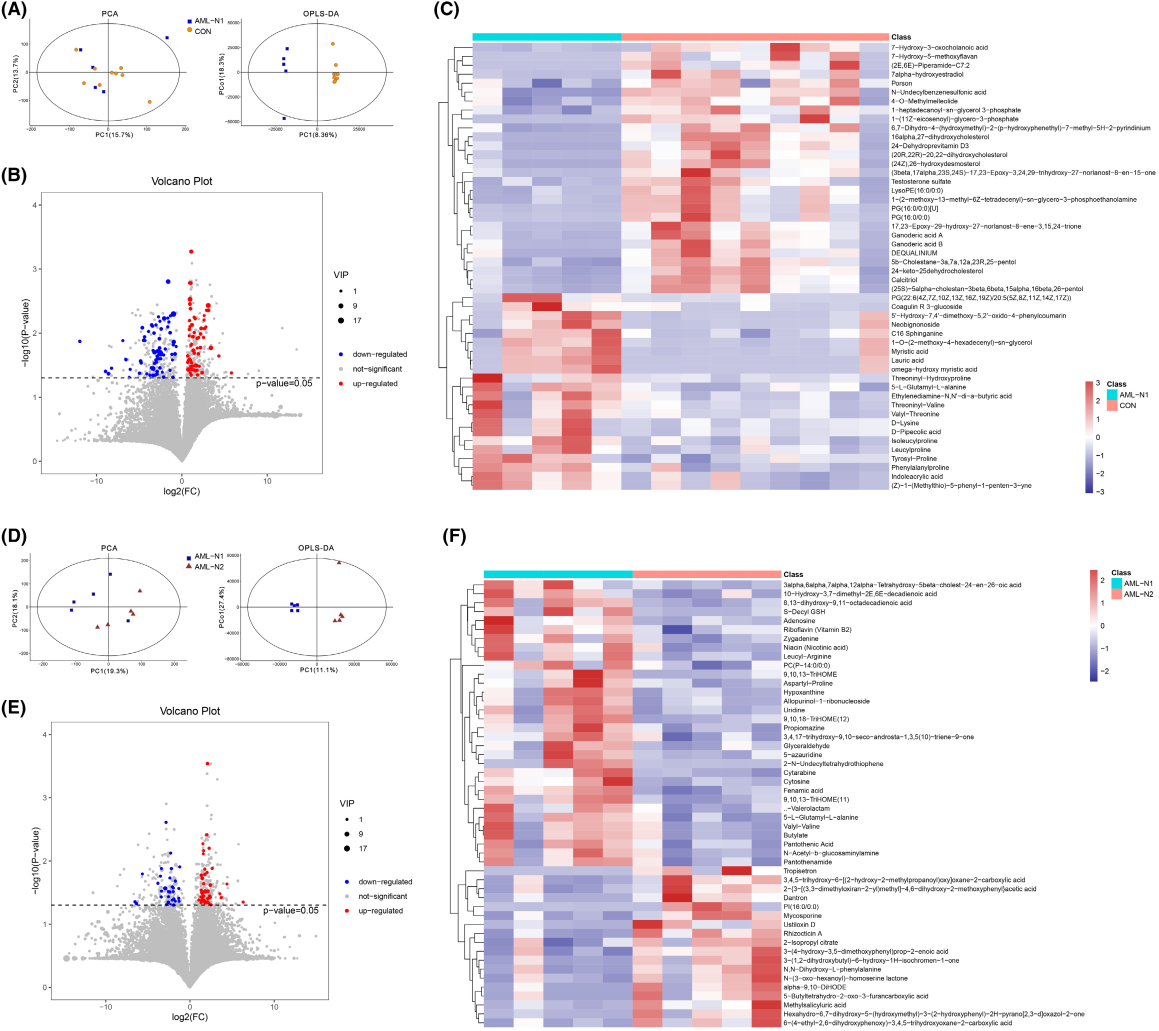
Multivariate statistical and differential fecal metabolites analysis. (A) Unsupervised PCA analysis between AML_N1 and CON groups. (B) Supervised OPLS‐DA analysis between AML_N1 and CON groups. (C) Hierarchical clustering of top 50 differential fecal metabolites between AML_N1 and CON groups. (D) Unsupervised PCA analysis between AML_N1 and ANL_N2 groups. (E) Supervised OPLS‐DA analysis between AML_N1 and ANL_N2 groups. (F) Hierarchical clustering of top 50 differential fecal metabolites between AML_N1 and ANL_N2 groups.

All of the fecal metabolite profiles in each group were obtained from LC–MS in both positive and negative ESI modes and qualitative, quantitative, and preprocessed using Progenesis QI V2.3. Thereafter, the metabolite profiles were imported into R software for metabolite profiles using ropls package. As shown in Figure 4, the results of unsupervised PCA analysis demonstrated the cross‐aggregated scatter plots between the compared groups (Figure 3A), indicating the stability and reliability of the data. The supervised OPLS‐DA analysis indicated that the scatter plots between the compared group were obviously distributed in different quadrants, revealing the remarkable difference in metabolic patterns between the compared groups. Thereafter, the differential metabolites were identified based on VIP ≥1, and *p* < 0.05, and all of the differentially expressed metabolites were displayed in Table S1. The corresponding volcano plots are shown in Figure 4B, where the red and blue points represented the upregulated and down‐regulated metabolites, respectively, demonstrating the significant difference between compared groups. Hierarchical clustering, performed to analyze the differential metabolites, clearly described the separation within the shortlisted metabolites in the respective groups and demonstrated the different patterns between AML patients and control individuals (Figure 4C). Figure 4D–F demonstrated the results of PCA and OPLS‐DA analysis, volcano plots of metabolites, and top 50 differential metabolites between AML patients and patients treated with chemotherapy, respectively, revealing the different metabolite patterns between respective groups. All of the differentially expressed metabolites between AML patients and patients treated with chemotherapy are displayed in Table S2.

### 3.3 Collection analysis between main differential metabolites and gut microbiota

**FIGURE 5:**
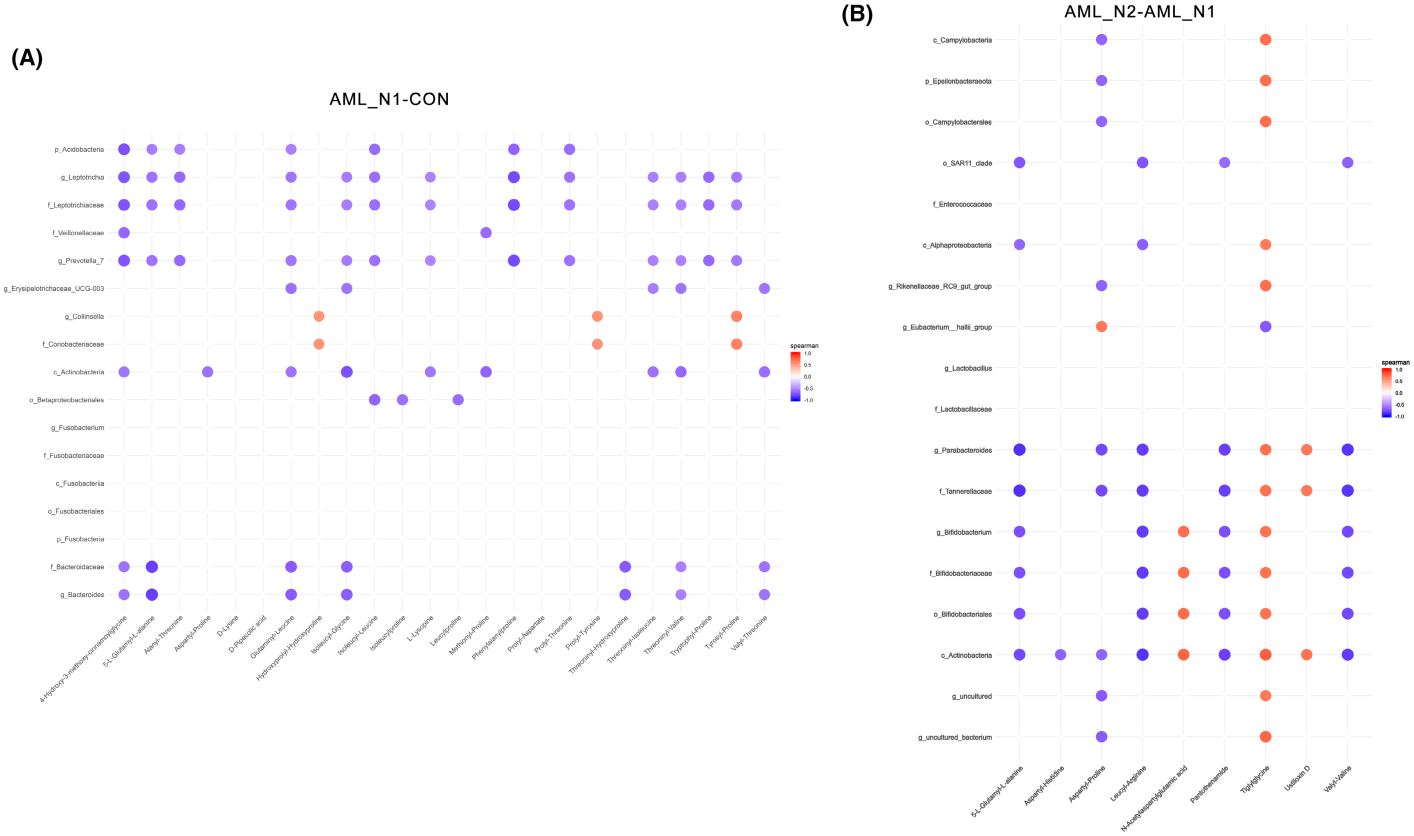
Correlation analysis between differential fecal amino acid metabolites and fecal bacterial biomarkers identified by LEfSe analysis. The color represents a significant correlation (*p* < 0.05), with orange representing a positive correlation and blue representing a negative correlation, and the color intensity represents the degree of association.

Interestingly, upon carefully analyzing the differential metabolites between groups, we found that, compared to patients in AML_N1 group, numerous differential expressed amino acids and analogs can be observed in individuals of both CON (Table 2) and AML_N2 groups (Table 3). Furthermore, we analyzed the correlations between bacteria biomarkers, identified by LEfSe analysis, and the differential amino acids and analogs using spearman association analysis, demonstrating that plenty of bacteria biomarkers were statistically correlated with multiple amino acids and analogs between the compared groups. Furthermore, we found that both *Collinsella* and *Coriobacteriaceae* demonstrate remarkable positive correlation with hydroxyprolyl‐hydroxyproline, prolyl‐tyrosine, and tyrosyl‐proline (Figure 5A,B).

**TABLE 2:** The differentially expressed amino acids and analogs in AML patients compared with control individuals.

No.	Metabolites	VIP	*p*‐Value	Up/down
1	d‐Lysine	5.352	0.032	Up
2	Threoninyl‐valine	4.950	0.019	Up
3	Phenylalanylproline	4.452	0.001	Up
4	Isoleucylproline	4.418	0.002	Up
5	Leucylproline	3.993	0.007	Up
6	d‐Pipecolic acid	3.800	0.032	Up
7	5‐l‐Glutamyl‐l‐alanine	2.945	0.004	Up
8	Tyrosyl‐proline	2.711	0.003	Up
9	Valyl‐threonine	2.609	0.020	Up
10	Threoninyl‐hydroxyproline	2.480	0.021	Up
11	Hydroxyprolyl‐hydroxyproline	2.125	0.033	Up
12	l‐Lysopine	2.057	0.010	Up
13	Glutaminyl‐leucine	1.925	0.023	Up
14	Threoninyl‐isoleucine	1.873	0.037	Up
15	Aspartyl‐proline	1.548	0.016	Up
16	Prolyl‐aspartate	1.538	0.042	Up
17	Isoleucyl‐glycine	1.527	0.045	Up
18	Alanyl‐threonine	1.438	0.027	Up
19	Tryptophyl‐proline	1.408	0.039	Up
20	Isoleucyl‐leucine	1.342	0.006	Up
21	Prolyl‐threonine	1.315	0.025	Up
22	4‐Hydroxy‐3‐methoxy‐cinnamoylglycine	1.292	0.045	Up
23	Methionyl‐proline	1.204	0.026	Up
24	Prolyl‐tyrosine	1.193	0.011	Up

**TABLE 3:** The differentially expressed amino acids and analogs in AML patients compared with AML patients treated with chemotherapy.

No.	Metabolites	VIP	*p*‐Value	Up/down
1	Valyl‐valine	4.693	0.044	Up
2	5‐l‐Glutamyl‐l‐alanine	2.853	0.017	Up
3	Leucyl‐arginine	2.120	0.039	Up
4	Ustiloxin D	1.494	0.037	Down
5	Pantothenamide	1.438	0.033	Up
6	Aspartyl‐proline	1.433	0.046	Up
7	Aspartyl‐histidine	1.152	0.044	Up
8	*N*‐acetylaspartylglutamic acid	1.136	0.012	Down
9	Tiglylglycine	1.123	0.027	Down

## 4 DISCUSSION

Gut microbiota was evidenced to show critical function in human health, and dysbiosis of intestinal flora was associated with pathological process of neurodegenerative diseases,^25^ colorectal cancer,^26^ fever duration in pediatric patients undergoing allogeneic hematopoietic stem cell transplantation,^27^ and hematological malignancies.^28^ As reviewed by Masetti et al., gut microbiota influenced the occurrence, treatment course, and associated complications of pediatric acute leukemia, implicating the regulating effect of gut microbiota in leukemia.^29^ In our current work, we preliminary investigated the changes of gut microbiota in AML patients compared to that in normal individuals. α‐diversity analysis showed that AML patients harbored less microbial communities, which was increased by chemotherapy. β‐diversity presented by the PCoA plot revealed the distinguished microbiota profiles among these three groups, which were consistence with previous report.^30^ Although numerous studies have demonstrated the decrease of diversity in AML patients treated with chemotherapy than that in newly diagnosed AML, the patients administrated with antibiotic prior to or during chemotherapy.^14^, ^31^ Antibiotics have been found to break the gut homeostasis and change the communities of gut microbiota, thereby decreasing microbial diversity, although gut microbiota itself has recover ability.^32^, ^33^ The reason that makes our current results difference may partial due to the absence of antibiotics. Additionally, the loss in diversity was not universal, and some patients actually gained diversity during chemotherapy,^34^ which is consistent with our present study. Further analysis showed that, at the phylum level, the ratio of *Firmicutes* to *Bacteroidetes* was increased in AML patients than that in normal individuals, indicating a microbial dysbiosis in AML patients,^24^, ^35^ which was slightly rescued by chemotherapy. LEfSe analysis suggested that *Collinsella* and *Coriobacteriaceae* were significantly enriched in newly diagnosed AML patients compared to that in control individuals, whereas *Eubacterium_hallii_group* was obviously enriched in AML patients compared with that in patients treated with chemotherapy. These results revealed the significant different profiles of gut microbiota derived from AML patients and patients treated with chemotherapy or healthy subjects. Similarly, the genera *Collinsella* was found to be enriched in several malignant tumors, such as colorectal cancer,^36^ gastric cancer,^37^ esophageal cancer,^38^ and hematological malignant.^39^

Emerging evidences have demonstrated that metabolites generated by gut microbiota had the ability to modulate the physiological functions of the host. As reported by Zarour, gut microbiota‐derived metabolites involve in immunoregulation in tumor.^40^ Gut microbe‐derived metabolite trimethylamine N‐oxide promotes antitumor immunity to pancreatic ductal adenocarcinoma, thereby strengthening response to anti‐PD1 in patients.^41^ Wang et al. have also reported that microbiome accelerates AML progression in a metabolite‐dependent manner.^42^ However, expanded studies are necessary to complement the role of gut microbes and metabolites in AML. Herein, we further analyzed the fecal metabolite profiles of AML patients treated with/without chemotherapy and normal populations, revealing the remarkable difference between groups in metabolic patterns. Interestingly, we found that compared to patients in AML_N1 group, numerous unique differential amino acids and analogs could be observed in individuals of both CON and AML_N2 groups. Nutrients amino acids is a group of fundamental sources for the maintenance of homeostasis and cellular processes in all organisms.^43^ Disturbance of amino acids and derivatives metabolism are associated with diverse disease including cancer through regulating various biological progress such as cell survival, immune response, epigenetics and redox.^44^ As found by Jones et al., the leukemia stem cells survival are uniquely dependent on amino acids, pharmacological suppression of amino acid metabolism impedes oxidative phosphorylation and contributes to cell death.^45^ Amino acid metabolism have been considered as promising targets for cancer therapy.^46^, ^47^, ^48^ In addition, evidences have demonstrated the complexity interplay between gut microbiota and amino acids in the pathophysiological processes of many diseases. For instances, Yan et al. have suggested that gut microbiota might be associated with Parkinson's disease by altering branched‐chain amino acid metabolism.^49^ Arnoriaga‐Rodriguez et al. have found that gut microbiota impair memory through regulating aromatic amino acids, specifically in subjects with obesity.^50^ In this work, spearman association analysis showed that plenty of bacteria biomarkers distinguished by LEfSe are statistically correlated with differential amino acids and analogs. It is worth noting that the bacteria biomarkers *Collinsella* and *Coriobacteriaceae* in AML patients showed remarkable positive correlation with Hydroxyprolyl‐Hydroxyproline, Prolyl‐Tyrosine, and Tyrosyl‐Proline. Overall, our data revealed that the alterations of gut microbiota correlate to fecal metabolites.

## 5 CONCLUSION

In conclusion, our 16S rRNA sequencing demonstrated the difference fecal microbiome profiles among control individuals, AML patients, and AML patients treated with chemotherapy. Further metabolomics combined analysis showed that gut microbiota may affect the pathophysiology of AML by regulating amino acids and their derivatives. The limitations of this work are that, on one hand, the sample size is too small, and on the other hand, we only suggested the correlation between intestinal microbes and amino acids in AML patients, the specific regulatory relationship between them need further elucidate. In addition, having a sample only after 7 days of treatment is less informative.^51^, ^52^ The follow‐up study will focus on expanding the sample size and extend sampling time to validate our current conclusion and to investigate the specific regulatory relationship between the gut microbiota and amino acid metabolites in AML patients, which may, in turn, guide medication. Overall, our present study implies the application value of 16S rRNA sequencing in the clinical diagnosis and treatment guidance of AML, and provides the possibility of AML treatment by gut‐microbiome–metabolome axis in the further.

## AUTHOR CONTRIBUTIONS

**Jing Xu:** Conceptualization (equal); data curation (equal); investigation (equal); methodology (equal); writing – original draft (equal). **Yong Kang:** Conceptualization (equal); data curation (equal); investigation (equal); methodology (equal); writing – original draft (equal). **Yan Zhong:** Conceptualization (equal); data curation (equal); formal analysis (equal); investigation (equal); writing – original draft (equal). **Wencan Ye:** Data curation (equal); formal analysis (equal). **Tianle Sheng:** Data curation (equal); formal analysis (equal). **Qingming Wang:** Data curation (equal); formal analysis (equal). **Jifu Zheng:** Data curation (equal); formal analysis (equal). **Qiuyue Yang:** Formal analysis (equal). **Ping Yi:** Formal analysis (equal). **Zhenjiang Li:** Conceptualization (equal); funding acquisition (equal); project administration (equal); supervision (equal); writing – review and editing (equal).

## CONFLICT OF INTEREST STATEMENT

The authors have no potential conflict of interest to declare.

## ETHICS STATEMENT

This study was approved by the Ethics Committee of The Second Affiliated Hospital of Nanchang University and all the patients provided informed consent (approval number: 2018‐098).

## Supporting information


Table S1.

Table S2.

## Data Availability

The data that support the findings of this study are available from the corresponding author upon reasonable request.
